# ﻿Description of a new species of black coral in the family Aphanipathidae (Anthozoa, Antipatharia) from Puerto Rico

**DOI:** 10.3897/zookeys.1173.104141

**Published:** 2023-08-02

**Authors:** Jeremy Horowitz, Dennis M. Opresko, María del P. González-García, Andrea M. Quattrini

**Affiliations:** 1 Department of Invertebrate Zoology, Smithsonian Institution, National Museum of Natural History, Washington, DC, USA National Museum of Natural History Washington, DC United States of America; 2 Department of Marine Sciences, University of Puerto Rico, Mayagüez, Puerto Rico University of Puerto Rico Mayagüez Puerto Rico

**Keywords:** *Aphanipathes*, molecular phylogenetics, morphology, targeted capture, taxonomy, ultraconserved elements

## Abstract

Black corals (Anthozoa: Antipatharia) are an anthozoan lineage in the class Hexacorallia that occur across a wide range of habitats from the tropics to the poles and from surface waters to depths deeper than 8000 m. A new species of black coral, *Aphanipathespuertoricoensis***sp. nov.**, collected with a remotely operated vehicle 357 m deep off Puerto Rico is recognized in the family Aphanipathidae. The new species is characterized by very long and loosely coiled primary branches and up to 0.5 mm tall spines with as many as 40 or more small conical tubercles. A phylogeny composed of 13 taxa that are closely related to the new species was reconstructed from 793 nuclear loci to show their systematic relationships. Our study integrated morphological and genomic data to show that this new species is distinct from other species in the genus *Aphanipathes.* Furthermore, our results add to the growing knowledge of black coral diversity, while further demonstrating the need for exploration in deep waters of the Caribbean Sea.

## ﻿Introduction

Black corals are an order in the sub-phylum Anthozoa (McFadden et al. 2022), which consists of 301 currently accepted species (WoRMS 2023) that are found in all oceans from just below the surface down to depths greater than 8000 m. Black corals are an enigmatic group due to logistical challenges associated with collecting species, most of which live deeper than 50 m depth (Molodtsova et al. 2023), and because many species are known from only one or a few specimens. Also, single-locus and full mitochondrial genomes lack the required phylogenetic resolution to resolve relationships between closely related species and genera. However, utilization of high-throughput genomic data and more efficient sampling techniques, like Remotely Operated Vehicles (ROVs), can be effectively used to support new species descriptions and taxonomic placements within the order (Horowitz et al. 2022).

An expedition in April 2022 south of Puerto Rico in the Caribbean Sea collected biological specimens with a ROV to document deep-water biodiversity. This region was targeted because deep waters in the area remain poorly characterized, and there is interest in documenting essential fish habitat as regional fisheries are extending into deeper waters (FAO 2015). During this expedition, a fragment of a one-meter-tall black coral was collected with an ROV at a depth of 357 m from Guayanilla Canyon. The specimen had long branches that spiraled at their distal ends, wide distal branch angles, and a high density of tubercles on the skeletal spines, presenting morphological features of two different genera: *Aphanipathes* Brook, 1889 and *Anozopathes* Opresko & Bo, 2021. Herein, we integrate morphological and genomic evidence to systematically describe and place the new species in *Aphanipathes*.

## ﻿Material and methods

### ﻿Specimen collection

**Figure 1.**
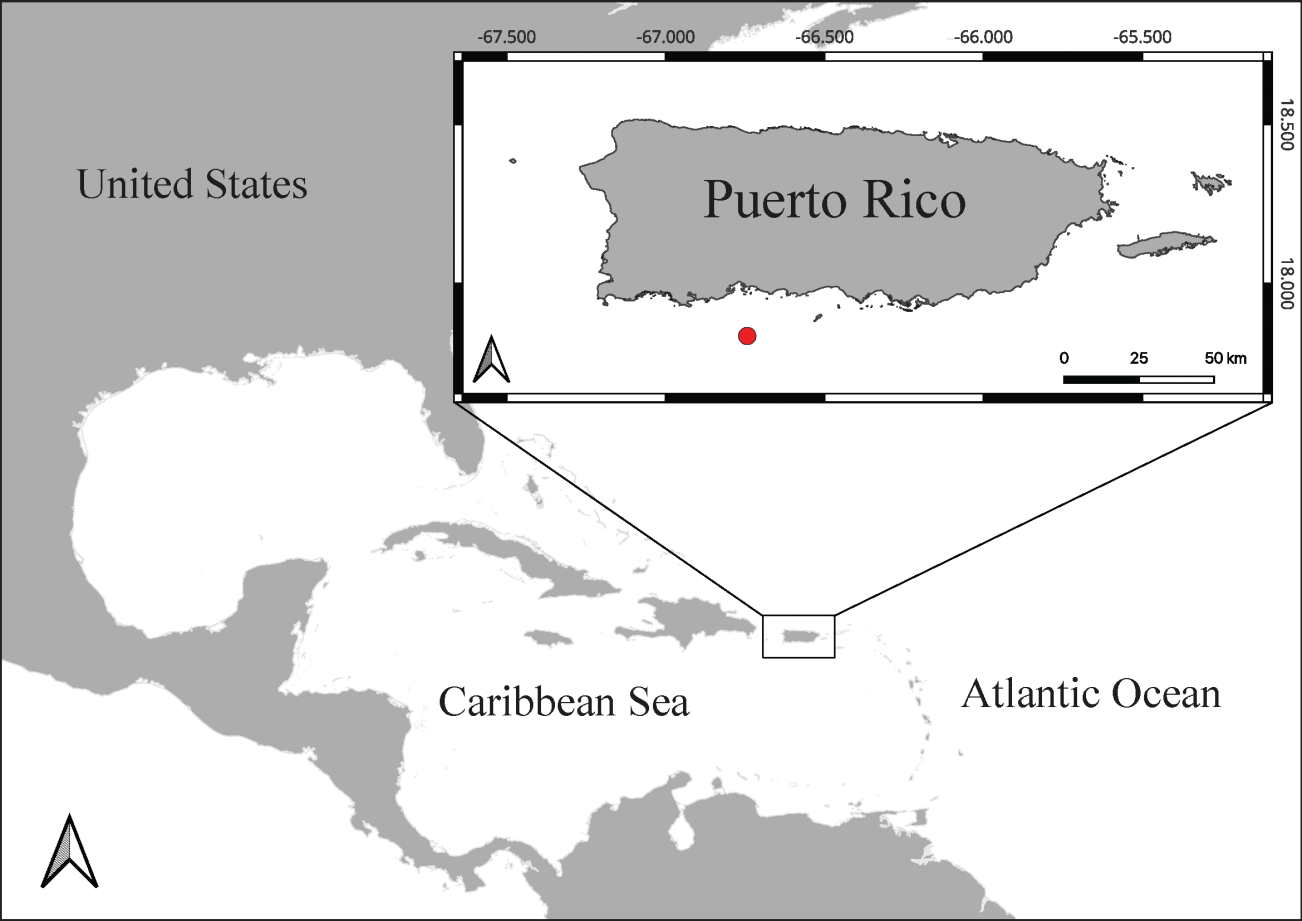
Location (denoted by a red circle) where *Aphanipathespuertoricoensis* sp. nov. was collected.

The new species was collected from Guayanilla Canyon off Puerto Rico in the Caribbean Sea at a depth of 357 m during an expedition of the NOAA ship “Nancy Foster” entitled: Illuminating Pelagic and Benthic Biodiversity in Deep Waters of Puerto Rico (Fig. 1). The new species was imaged with high-resolution video and then subsampled by cutting a ~35 cm distal section of branch using a coral cutter on the manipulator arm of the ROV Global Explorer. The specimen was curated and deposited in the collections of the National Museum of Natural History (NMNH), Smithsonian Institution, Washington DC, with the catalog number USNM 1660436. Specimens from the collections of the Natural History Museum, London, are indicated with the prefix “NHMUK”. Molecular data were deposited in the short read archive (SRA) of the National Center for Biotechnology Information (https://www.ncbi.nlm.nih.gov/). Metadata for all specimens included in this study are detailed in Suppl. material 1.

### ﻿Morphological analyses

Spine characteristics were imaged with a Zeiss Evo MA15 scanning electron microscope. Spine height was measured as the distance from the spine tip to the middle of the base of the spine. Polyp and branch characteristics were measured with a dissecting microscope and terminal branch diameter was measured near the base of the branch. This specimen was identified as “new to science” based on differences between the specimen and material of other species in the genus (Gray 1857; Brook 1889; Opresko 2004; Opresko et al. 2016, 2021).

### ﻿Molecular analyses

DNA extractions of the new species along with *Aphanipathespedata* (Gray, 1857) (USNM 1410008) and *Anozopathespalauensis* Opresko & Brugler, 2021 (USNM 1007104) were performed using the DNeasy Blood and Tissue Kit (Qiagen, Germany) following the manufacturer’s protocol. The DNA was cleaned with a Qiagen Power Clean Pro kit and concentrations were estimated using a Qubit 4 Fluorometer (Invitrogen, US). For the new species, DNA was sheared using a QSonica Inc Sonicator Q800R to a target size range of 400–800 bp and then checked via gel electrophoresis on a 1.5% agarose gel; no shearing was performed for the two additional USNM samples. Following shearing, DNA libraries were prepared with the Kappa Hyper Prep protocol using a ½ reaction with iTruSeq adapters and dual indexes following Quattrini et al. (2018). For the new species of *Aphanipathes*, paired-end sequencing (150 bp) was performed on a full lane of a MiSeq shared with two other samples in the Laboratories of Analytical Biology at the NMNH. The USNM samples were sequenced on a NovaSeq6000 at the Oklahoma Medical Research Foundation Genomics Facility with other samples to obtain ~10M total paired end (PE) reads (150 bp) per sample. All lab work was conducted in the Laboratories of Analytical Biology at the NMNH.

### ﻿Phylogenetic analyses

Ultraconserved elements (UCE) and exon nuclear loci were bioinformatically obtained from the high-throughput sequencing data. First, raw reads were trimmed using Trimmomatic v. 0.35 (Bolger et al. 2014) and then assembled using Spades v. 3.15 (Prjibelski et al. 2020). Then, UCE and exon loci were extracted using the hexacoral-v2-baitset (Cowman et al. 2020), following the Phyluce pipeline (https://phyluce.readthedocs.io/en/latest/tutorials/tutorial-1.html) (Faircloth 2016) with some modifications such as minimum-identity and minimum-coverage thresholds set to 70%. These data were then combined with existing UCE and exon loci extracted from 10 previously published black corals (Quattrini et al. 2020; Horowitz et al. 2022). All loci were edge trimmed and aligned with MAFFT v. 7.130 (Katoh and Standley 2013). Then, phyluce_align_get_only_loci_with_min_taxa was used to obtain all loci with 60% taxon-occupancy, which were then concatenated using phyluce_align_concatenate_alignments.

Phylogenomic inference was conducted on the concatenated dataset of 13 species using maximum likelihood analysis in IQTree v. 2.1 (Minh et al. 2020). A partitioned analysis (Chernomor et al. 2016) was conducted on the dataset using the best model for each locus [-m TESTMERGE (Kalyaanamoorthy et al. 2017)]. Ultrafast bootstrapping [-bb 1000 (Hoang et al. 2018)] and the Sh-like approximate likelihood ratio test [-alrt 1000 (Anisimova et al. 2011)] were also selected. All analyses were run on the Smithsonian’s High-Performance Computing Cluster (doi.org/10.25572/SIHPC), except the phylogeny was plotted in FigTree v. 1.4.4.

## ﻿Results

### ﻿Taxonomic results

**Family Aphanipathidae Opresko, 2004**

#### ﻿Genus Aphanipathes

Taxon classificationAnimaliaAntipathariaAphanipathidae

Brook, 1889

56FE208F-2A0F-514C-A6EB-5E8D62A00E1A

##### Diagnosis (emended).

Colony sparsely to densely, uniserial or irregularly branched, with elongate, straight, curved, or coiled, often ascending branches. Spines maximum 0.5 mm tall with pronounced tubercles. Polyps 1–2 mm in transverse diameter, with three to eight polyps per centimeter.

##### Type species.

*Aphanipathessarothamnoides* Brook, 1889.

##### Type locality.

Vanuatu.

##### Remarks.

Brook (1889) erected the genus *Aphanipathes* to include the following five new species: *Aphanipathessarothamnoides* Brook, 1889, *Aphanipathesverticillata* Brook, 1889, *Aphanipathesalata* Brook, 1889, *Aphanipathesbarbadensis* Brook, 1889 and *Aphanipathescancellata* (Brook, 1889), as well as eleven previously described nominal species: *Antipatheseupteridea* Lamouroux, 1824; *Antipathessalix* (Pourtalès, 1880); *Antipathesrigida* Pourtalès, 1880; *Antipathesfruticosa* Gray, 1857; *Antipathespedata* Gray, 1857; *Antipathespennacea* Pallas, 1766; *Antipathesfilix* Pourtalès, 1867; *Antipatheswollastoni* Gray, 1857; *Antipatheshumilis* Pourtalès, 1867; *Antipathesthyoides* Pourtalès, 1880; and *Antipathesabietina* Pourtalès, 1874. The genus was revised to include only *A.sarothamnoides* (the type species) and *A.salix*, *A.pedata* and *A.verticillata*. Since then, a fifth species, *Aphanipathesflailum* Horowitz, 2022, has been added to the genus. All five species are branched, have polypar spines twice as large as the abpolypar spines, and possess distinct tubercles on the surfaces of polypar and abpolypar spines (Table 1). These species differ based on the density of tubercles on a visible lateral view of a given spine where *A.salix* has about five tubercles (which are also more knob-like than any other aphanipathids), *A.flailum* has about eight to 12 tubercles, *A.sarothamnoides* has about 15 tubercles, and *A.pedata* and *A.verticillata* have as many as 30 tubercles. *Aphanipathesverticillata* also has spines that form distinct verticils while all other species have spines in horizontal rows that form spirals. The phylogeny reconstructed in Horowitz et al. (2022) suggests that *A.verticillata* might be more closely related to the genus *Pseudocirrhipathes*; *A.salix* has never been sequenced and the differences in the ornamentation on the spines could suggest that it belongs to a different genus, but confirmation requires comparison of molecular data from holotype or topotype material of each species in the genus to formally revise the group.

**Table 1.** Comparison of species in the genus *Aphanipathes*.

	Growth form	Terminal branchlet length (cm)	Branchlet density per 5 cm	Number of branch orders	Distal branch angle	Polypar/ abpolypar spine heights (mm)	*Tubercle density on polypar spine (per side view)	Polyp in transverse diameter (mm)	Polyp density (per cm)
*Aphanipathespuertoricoensis*	Multi-plane	> 20	1–2	2–3	90	0.5/0.3	20–40	1.1–1.6	6–8
*Aphanipathesverticillata*	Multi-plane	10	4–8	10 +	45–60	0.28/0.14	20–30	1.4	6–7
*Aphanipathessalix*	Fronds	3	1–2	3–6	30–45	0.22/0.13	5	1.2	6
*Aphanipathespedata*	Fronds	10	5–8	8 +	30–45	0.4/0.22	30	1.2–1.7	5–8
*Aphanipathessarothamnoides*	Fronds	10	1–2	3–4	30–45	0.2/0.1	12–15	1.3	6
*Aphanipathesflailum*	Fronds	10	1–2	6	30	0.12/0.09	8–12	1.8–2.0	3–4

* Including tubercles on the distal and proximal edges of spines.

#### ﻿ Aphanipathes puertoricoensis

Taxon classificationAnimaliaAntipathariaAphanipathidae

Horowitz & Quattrini sp. nov.

B1AFC2F8-5343-596E-9A8A-7A5604DE68EE

https://zoobank.org/5AD4DED2-215F-4BC5-AB58-2739FD4D0183

**Figure 2.**
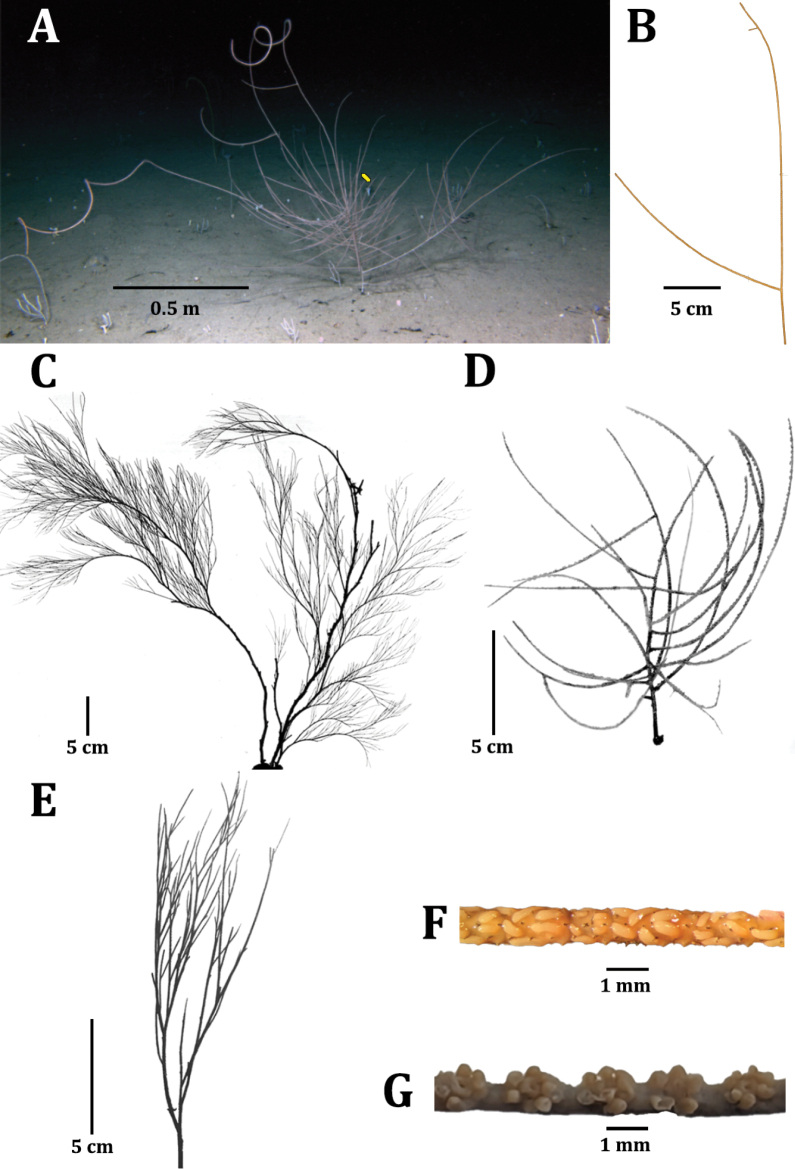
Comparison of species of *Aphanipathes* and *Anozopathes*: **A***Aphanipathespuertoricoensis* sp. nov., yellow line indicates from where holotype fragment USNM 1660436 was collected **B***Aphanipathespuertoricoensis* sp. nov. holotype collected fragment (USNM 1660436) **C***Aphanipathespedata* holotype (NHMUK 1843.2.6.105) **D***Anozopathespalauensis* holotype (USNM 1007104) **E***Aphanipathessarothamnoides* part of holotype (NHMUK 1890.4.9.5) **F** polyps of *Aphanipathespuertoricoensis* sp. nov. holotype (USNM 1660436) **G** polyps of *Anozopathespalauensis* holotype (USNM 1007104).

**Figure 3.**
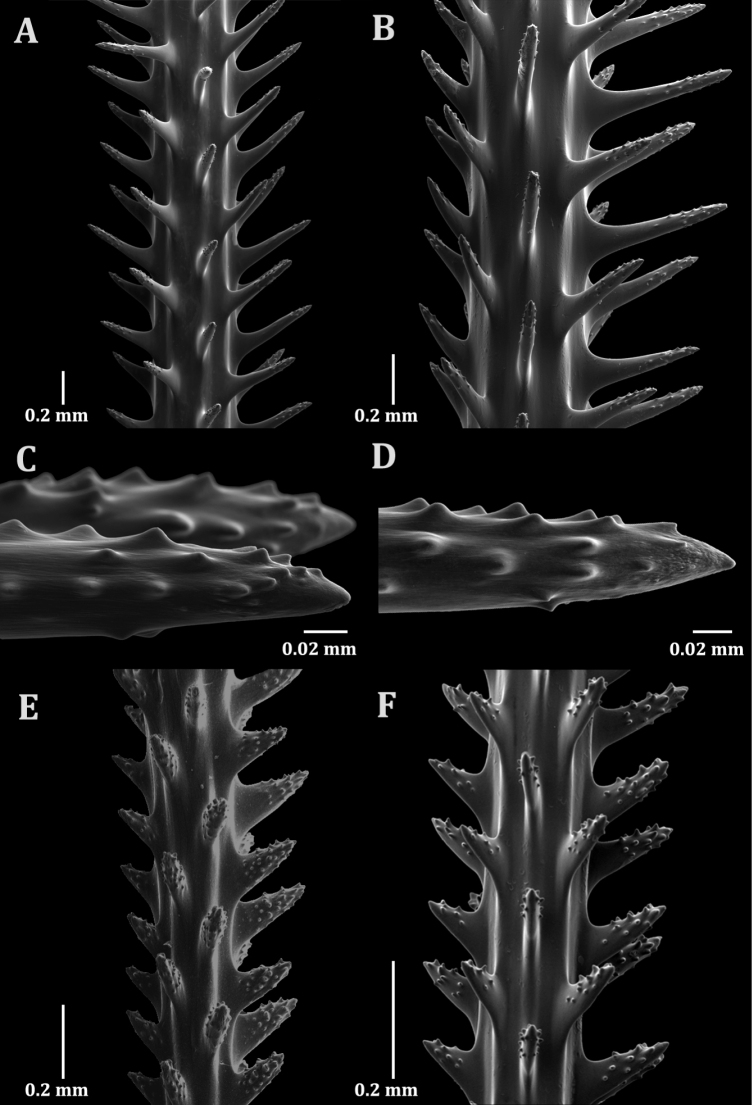
Skeletal spines of *Aphanipathespuertoricoensis* sp. nov. holotype (USNM 1660436) (**A–D**) and *Aphanipathespedata* (**E, F**): **A** spines on branch 0.45 mm in diameter **B** spines on branch 0.45 mm in diameter **C** small conical tubercles on spine tips with faint striations between tubercles **D** small conical tubercles on spine tip with slight papillae between tubercles **E***Aphanipathespedata* holotype (NHMUK 1843.2.6.105), spines on branch about 0.22 mm in diameter **F**Aphanipathescf.pedata (USNM 1410008), spines on branch 0.16 mm in diameter.

**Figure 4.**
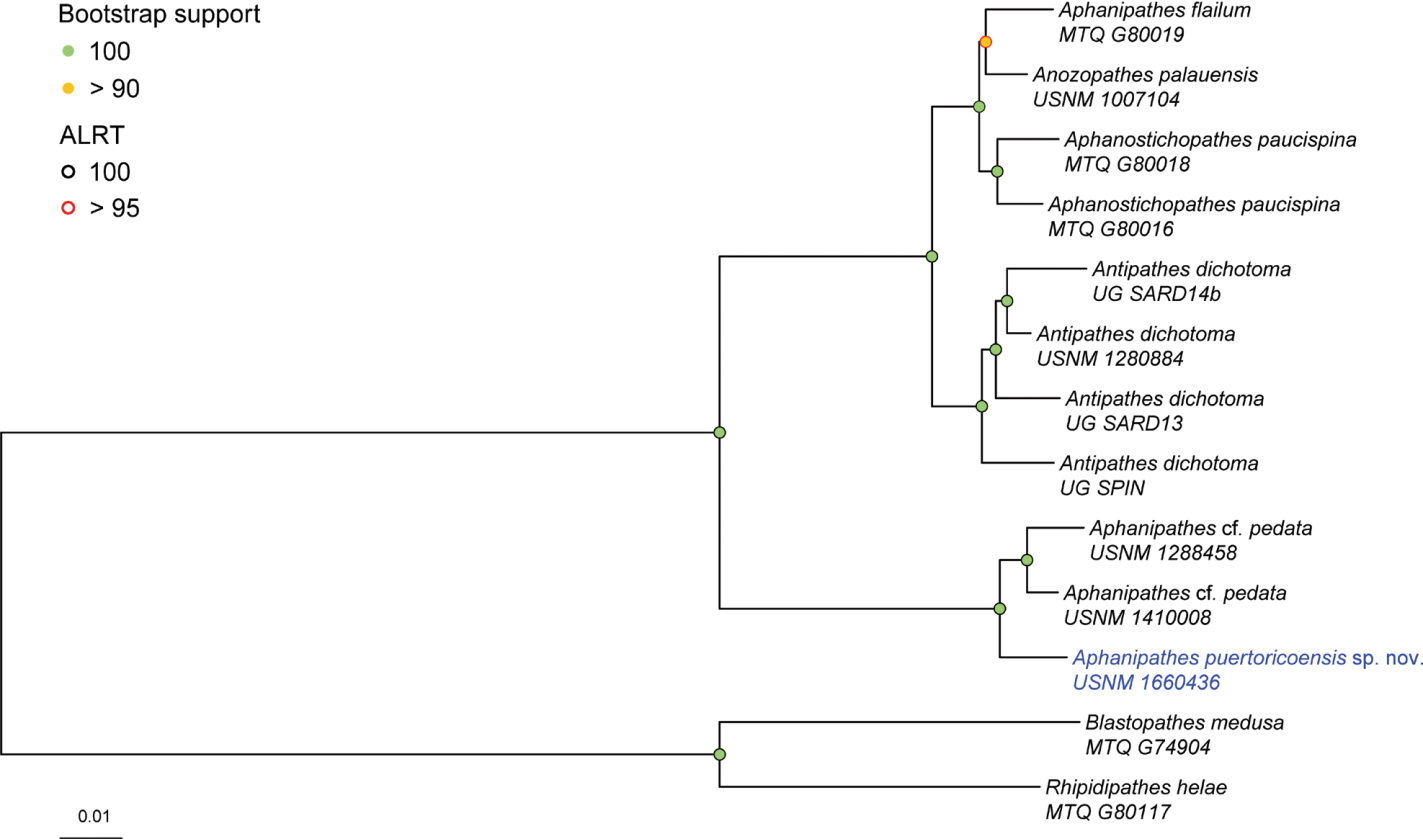
Maximum likelihood phylogeny of antipatharians closely related to the new species based on a 60% complete matrix containing 793 loci. Taxon in blue represents the holotype of the new species. The phylogeny was rooted to the *Rhipidipathes* and *Blastopathes* clade.

Figs 1 , 2 , 3 , 4 ; Table 1 ; Suppl. materials 1 , 2

##### Material examined.

***Holotype***: USNM 1660436 (USNM SEM stubs 530–532), Guayanilla Canyon, off Puerto Rico, station GEX-22-04, 17°54'17.80"N, 66°43'18.78"W, NOAA Ship Nancy Foster, Expedition: Illuminating Pelagic and Benthic Biodiversity in Deep Waters of Puerto Rico, 357 m depth, coll. A.M. Quattrini, A.G. Collins & E.E. Cordes, 12 April 2022.

##### Type locality.

Guayanilla Canyon, Puerto Rico, Caribbean Sea, 357 m depth.

##### Diagnosis.

Tall, sparsely branched colony with two and rarely three orders of branches. Stem 0.2 m tall, first order branches over 1 m long, distally slightly curved or coiled, and arranged irregularly around stem; second order branches short and sparsely occurring; third order branches rare. Distal branch angles usually close to 90°. Spines form a distinct quincunx pattern with six axial rows of spines counted in one view. Polypar spines up to 0.5 mm tall, possess 20 to 40 or more small conical tubercles visible in lateral view that extend from the tip halfway to the base. Abpolypar spines up to 0.32 mm tall, possess 10 to 20 small conical tubercles visible in lateral view that extend from the tip halfway to the base. Polypar and abpolypar spines spaced 0.47 to 0.57 mm apart with three spines per mm in each row. Polyps 1.1 to 1.6 mm in diameter, arranged in a single series, with six to eight polyps per cm.

##### Description of holotype.

The holotype (USNM 1660436) is a fragment from the whole colony (Fig. 2A) that is 0.35 m tall and includes a first order and two second order branches (Fig. 2B). The diameter near the base of the second order branch is about 1 mm including spines.

The spines are laterally compressed and arranged in a quincunx pattern with five to six longitudinal rows in one view and three spines per cm counted in one longitudinal row (Fig. 3A, B). On a section of branch 0.3 mm in diameter, the polypar spines are about 0.35 mm tall and the abpolypar spines are 0.16 mm tall. On a section of a branch 0.45 mm in diameter the polypar spines are about 0.5 to 0.6 mm tall and the abpolypar spines are about 0.32 mm tall (Fig. 3B). Polypar spines have a distal angle of about 75° and abpolypar spines have a distal angle of about 45° (Fig. 3A, B). The spines have conical tubercles on their surface extending from the apex to about midway down the spine towards the base. Based on counts made on one lateral side of a spine and including those seen on the distal and proximal edges, most polypar spines have between 20 to 40 small conical tubercles while the abpolypar spines have about half as many tubercles (Fig. 3B). The conical tubercles are 0.007 mm tall and run at right angles to the direction of the spine. Within these rows about three tubercles can be counted over a 0.1 mm distance (Fig. 3C, D). Between the tubercles faint striations can be observed (Fig. 3C) and sometimes slight papillae or immature tubercles (Fig. 3D).

The polyps are arranged in a single row. In the preserved state the polyps are 1.1–1.6 mm in transverse diameter with an interpolypar space of about 0.15 mm, and there are six to eight polyps per cm (Fig. 2F). There is no notable difference in the size of the sagittal and lateral tentacles based on preserved material.

##### Description of colony from which holotype was collected.

The colony from which the holotype was collected was videotaped *in situ* and based on a screen shot from that video (Fig. 2A) the complete colony is approximated to be over 1 m tall and over 2 m wide including branches. The main stem is approximated to be 0.2 m tall and consists of approximately eight irregular rows of primary branches, spaced about 2 cm apart in a row. Primary branches are long and loosely coiled at their distal end, reaching lengths of over 1 m. Secondary branches are sparse and third order branches are rare.

##### Phylogenetic results.

We obtained between 2 to 15 million PE reads per specimen, which assembled into 54,513 to 287,021 contigs. The new *Aphanipathes* species had 5,400,183 reads and 262,434 assembled contigs. From the contigs, 1186 to 1609 UCE and exon loci were obtained per specimen. The 60% taxon-occupancy matrix included 793 loci that were concatenated into an alignment with a total length of 365,565 bp. Read and locus summary statistics are detailed in Suppl. material 2. The phylogeny (Fig. 4) was strongly supported, with 100% bootstrap and Sh-alrt values at every node but one. In addition, the topology is congruent with the phylogeny presented in Horowitz et al. (2022) and Opresko et al. (2021).

Based on specimens included, phylogenetic results place the new species sister to two specimens that most closely resemble *Aphanipathespedata* (Gray, 1857) from 229 m depth in the Gulf of Mexico (USNM 1288458) and 155 m depth from the Caribbean Sea (USNM 1410008) (Fig. 4). Unfortunately, both specimens are incomplete colonies, we lack molecular data for the *A.pedata* holotype, and spine comparisons are based on just one SEM of the holotype (Fig. 3E). For these reasons, we conservatively identify these two specimens as A.cf.pedata. We also lack molecular data for the type of the genus, *A.sarothamnoides*, which will be useful when reviewing the taxonomy of the genus.

##### Comparative diagnosis.

The new species has morphological features that fit the emended diagnosis of *Aphanipathes*, including a branched corallum, polypar spines being twice as tall as abpolypar spines, pronounced tubercles on skeletal spines, and small polyps between 1 and 2 mm in transverse diameter, reaching a density of eight polyps per cm. In addition to phylogenetic affinity, the new species shares a high density of tubercles on the surfaces of spines, being greater than 20 counting all tubercles on the visible side of a spine, and similar sized polyps with *A.pedata* (Fig. 3E). However, the new species is different from *A.pedata* by having three-dimensional branching (*A.pedata* has branches arranged uniserially, forming fronds, larger terminal branchlets (> 20 cm vs 10 cm), wider terminal branchlet distal angles (90° vs 30–45°), and lower branch complexity (two vs eight or more orders of branches) (Fig. 2A–C). The new species also has tubercles that only extend midway down the spine and are generally less pronounced (Fig. 3B) compared to *A.pedata*, which has more pronounced tubercles present from the tip down to the bottom third or almost to the base of each spine (Fig. 3E, F). The new species also has spines that are distinctly thinner on their distal ends compared to the type of *A.pedata* and two A.cf.pedata specimens (Fig. 3A, B and Fig. 3E, F).

The new species is also different from the other species in *Aphanipathes* by having longer terminal branchlets (> 20 cm vs 10 cm or less), wider terminal branchlet distal angles (90° vs 30–60°), and is mostly branched to the second order compared to three or more orders in *A.sarothamnoides* (Fig. 2E), as many as six orders in *A.salix* and *A.flailum*, and ten or more orders in *A.verticillata*.

The new species has a branching pattern that is like *Anozopathes* Opresko & Bo, 2021 (Fig. 2D). However, the new species is a much larger colony compared to other *Anozopathes* due to branch lengths > 1 m vs max 0.18 m long, resulting in approximate colony widths and heights of 2 m (Fig. 2A) vs 0.16 m in *Anozopathespalauensis* Opresko & Brugler, 2021 (Fig. 2D) and 0.36 m in *Anozopatheshawaiiensis* Opresko & Bo, 2021. The new species also possesses a greater abundance of conical tubercles on the spines compared to *Anozopathes* (20–40 vs six to eight). Lastly, the new species has smaller polyps than *Anozopathes* (about 1.3 vs 2 mm in *Ano.palauensis* and 3.6 mm in *Ano.hawaiiensis*) and greater polyp density per cm (6–8 vs 3–5 in *Ano.palauensis* and 4–5 mm in *Ano.hawaiiensis*) (Fig. 2F, G).

##### Etymology.

The species name “puertoricoensis” is based on the type locality.

##### Distribution.

Known only from Guayanilla Canyon, Puerto Rico, Caribbean Sea; 357 m depth.

## ﻿Discussion and conclusion

### ﻿Current taxonomic challenges

The reconstructed phylogeny reveals that *Aphanipathes* is polyphyletic. However, *Aphanipathessarothamnoides*, the type of the genus by subsequent designation, has yet to be included in phylogenomic analyses, which makes a formal review of the genus difficult. In addition, the phylogeny revealed that *Antipathesdichotoma* Pallas, 1776, which is the type species of the Antipathidae, is more closely related to species in the Aphanipathidae than to other Antipathidae spp. (Brugler et al. 2013; Bo et al. 2018; Horowitz et al. 2022). Furthermore, *Anozopathes* and *Aphanostichopathes* Bo & Opresko, 2021 are two genera that have distinct tubercles on their skeletal spines like other aphanipathids, but differ in that *Anozopathes* has long stem-like branches and *Aphanostichopathes* is unbranched. However, the newly constructed phylogeny shows that both genera form a group with *Aphanipathesflailum* Horowitz, 2022, which does not have stem-like branches and is much more densely branched than *Anozopathes*. Another challenge is that Horowitz et al. (2022) found *Aphanipathesverticillata* Brook, 1889 has greater genetic affinity to the genus *Pseudocirrhipathes* Bo & Bavestrello, 2009 than *Aphanipathes*. Therefore, a formal review with integrated morphological and molecular data of holotype or topotype specimens representing species in these genera is required to resolve these taxonomic issues.

### ﻿Informed taxonomic decisions from multiple lines of evidence

The inclusion of molecular data in taxonomic studies is challenging paradigms about which morphological features are diagnostic and homologous (traits passed down from a common ancestor) and which are analogous (similar traits independently evolved). For example, it was once thought that an unbranched morphology was homologous; however, recent studies have illuminated that this trait is analogous, occurring in at least two divergent families: Antipathidae (*Stichopathes* Brook, 1889, *Cirrhipathes* de Blainville, 1830 and *Pseudocirrhipathes* Bo et al., 2009) and Aphanipathidae (*Aphanostichopathes*) (Brugler et al. 2013; Barrett et al. 2020; Opresko et al. 2021; Terrana et al. 2021; Horowitz et al. 2022). In the present study, we describe a new species that has long, stem-like branches like *Anozopathes* and *Blastopathes* Horowitz, 2020; however, the phylogenetic reconstruction shows that the new species has the greatest phylogenetic affinity to *Aphanipathes*, a genus without a species with these branching characteristics, and *Anozopathes* and *Blastopathes*, which group with species that form flabellate colonies that lack stem-like branches (Fig. 4). Therefore, general branching characteristics can often be analogous. These findings highlight the importance of using integrated morphological and molecular data when making taxonomic decisions to identify and incorporate homologous features in the taxonomic decision-making process. These findings also signify the need for an integrated taxonomic review of the order, and subsequent revisions based on holotype and topotype material.

### ﻿Continued exploration in the deep Caribbean Sea

Deep-water (> 200 m) biodiversity off Puerto Rico, and broadly throughout the Caribbean Sea, remains poorly characterized. Yet, this area is critical to understanding the biogeographical patterns and connectivity of corals in the North Atlantic Ocean. Our study showcases the necessity of future exploration in this region. From just one expedition that included only five ROV dives with targeted collections, at least one new coral species was found. Discovery of the new species with morphological features similar to multiple genera demonstrates how morphological features can be misleading and highlights the importance of using integrated approaches to describing new species. It is important to continue collecting in deep waters of the region as it will lead to a better understanding of species’ ranges, and improved estimates of regional biodiversity, both of which are required to make informed conservation decisions that mitigate drivers of biodiversity decline.

## Supplementary Material

XML Treatment for Aphanipathes

XML Treatment for Aphanipathes puertoricoensis

## ﻿Additional information

### Author ORCIDs

Jeremy Horowitz https://orcid.org/0000-0002-2643-5200

### Data availability

All of the data that support the findings of this study are available in the main text or Supplementary Information.

## Supplementary materials

Supplementary material 1 Metadata for specimens included in this study Data type table (Excel spreadsheet) Explanation note **table S1**: Specimen data including museum registration ID, family ID, genus ID, species ID, location and depth of collected specimens, and date collected. https://binary.pensoft.net/file/887389This dataset is made available under the Open Database License (http://opendatacommons.org/licenses/odbl/1.0/). The Open Database License (ODbL) is a license agreement intended to allow users to freely share, modify, and use this Dataset while maintaining this same freedom for others, provided that the original source and author(s) are credited.Jeremy Horowitz, Dennis M. Opresko, María del P. González-García, Andrea M. Quattrini

Supplementary material 2 Read and locus summary statistics for specimens included in the study Data type table (Excel spreadsheet) Explanation note **table S2**: Molecular data including the number of raw reads, assembly statistics, and NCBI information. https://binary.pensoft.net/file/887390This dataset is made available under the Open Database License (http://opendatacommons.org/licenses/odbl/1.0/). The Open Database License (ODbL) is a license agreement intended to allow users to freely share, modify, and use this Dataset while maintaining this same freedom for others, provided that the original source and author(s) are credited.Jeremy Horowitz, Dennis M. Opresko, María del P. González-García, Andrea M. Quattrini
